# Effect of COVID-19 and other determinants on the reduction of non-urgent emergency department access in North-East Italy: does supply affect demand?

**DOI:** 10.1007/s43999-025-00073-1

**Published:** 2025-09-01

**Authors:** Michele Gobbato, Federico Vola, Ivana Burba, Luca Lattuada, Laura Regattin

**Affiliations:** 1 ARCS – Azienda Regionale di Coordinamento per la Salute, Udine, Italy; 2 Fondazione Casa Cardinale Maffi ONLUS, Cecina, Italy; 3 Scuola Superiore Sant’Anna, Pisa, Italy; 4 Direzione Centrale Salute, Politiche Sociali e Disabilità - Regione Autonoma Friuli Venezia Giulia, Trieste, Italy

**Keywords:** COVID-19, Emergency departments, Non-urgent access, Healthcare demand, Friuli venezia giulia, Healthcare utilization, Geographic determinants

## Abstract

This study examines the impact of the COVID-19 pandemic on non-urgent emergency department (ED) utilizzation in Friuli Venezia Giulia, Italy, comparing the pre-COVID (2019) and COVID (2021) periods. A retrospective observational analysis at the municipal level assessed admission rates of non-urgent patients, identified by “white code” triage. Variables analyzed included driving time to the nearest ED, altitude, and the effect of closing first intervention points. Results showed a significant 31% reduction in non-urgent ED visits from 2019 to 2021, with a mean reduction of 37.8% in municipalities affected by ED closures. A multivariate linear model showed that increased driving time and higher pre-pandemic admission rates were both associated with greater reductions in access, while altitude showed a positive correlation with access rates. The findings suggest that service supply limitations, such as ED closures, significantly reduce non-urgent ED utilization, especially in lowland and hilly areas, but less so in mountainous regions.

**Supplementary Information:**

The online version contains supplementary material available at 10.1007/s43999-025-00073-1.

## Introduction

In Italy, as in many developed countries, a high number of non-urgent (inappropriate) patients seeking care in Emergency Departments (EDs) contributes to increased healthcare costs, longer waiting times, and greater pressure on healthcare services [1, 2]. Non-urgent visits to EDs are often indicative of limited confidence in primary care and a stronger reliance on hospital-based services [3]. The limited accessibility of primary care services, typically operating only during business hours, makes scheduling appointments difficult and drives some patients to seek care in EDs even for non-urgent conditions [4]. The proportion of non-urgent ED utilization varies between 20% and 40%, depending on the method used to measure it [3, 5, 6]. For example, a 2013 survey in France involving 29,407 patients found that 23.6% of ED visits were non-urgent based on an appropriateness score, 27% could have been managed by general practitioners, and 13% were deemed inappropriate based on resource utilization. Patients reported turning to EDs due to the unavailability of their general practitioner (GP) or because accessing ED services was quicker than securing a primary care appointment [6]. Non-urgent ED use is also associated with younger age groups and patients who use private transportation to reach the hospital [3]. Proximity to ED facilities, as well as socio-economic vulnerability, are additional factors influencing non-urgent visits [6].

In many developed countries, emergency departments (EDs) face sustained pressure from patients presenting with conditions that are not urgent or life-threatening. This phenomenon, often referred to as inappropriate or non-urgent ED use, is typically defined as access to emergency care for conditions that could be safely managed in primary care settings or delayed without immediate risk to the patient [1, 2]. In contrast, appropriate ED use refers to time-sensitive, severe, or complex medical cases requiring hospital-based diagnostics or interventions.

Understanding what drives non-urgent ED use is critical, as it contributes to ED overcrowding, increases healthcare costs, and diverts resources from patients with real emergencies [3, 4]. Research has shown that non-urgent ED use is influenced by both demand-side factors (e.g., patient behavior, socio-economic status, perceived urgency, availability of primary care) and supply-side factors (e.g., proximity to EDs, healthcare infrastructure, organization of regional services) [5–7].

Previous studies have demonstrated wide geographical variation in ED access, often unrelated to population health needs [8]. This suggests that structural, contextual, or cultural determinants—rather than clinical necessity—may influence access patterns. For instance, people living in rural or mountainous areas may rely more on EDs due to poorer access to general practitioners (GPs) or limited out-of-hours services. Altitude, in this context, can serve as a proxy for rurality and isolation, which may explain differences in ED-seeking behavior, especially when healthcare access is further constrained, as during the COVID-19 pandemic [9].

The pandemic created an unprecedented disruption in healthcare access and patient behavior, offering a unique opportunity to study how exogenous shocks affect non-urgent ED use. Lockdowns, fear of contagion, and the closure of minor emergency care facilities (e.g., first intervention points) may have reduced non-urgent ED access and highlighted how service availability shapes healthcare-seeking choices [10, 11]. However, it is unclear whether all populations responded similarly. For example, elderly patients with chronic conditions might have reduced their ED visits due to fear of infection, while parents of young children may have continued to rely on EDs as a primary source of reassurance or care.

Friuli Venezia Giulia (FVG) is a medium size region in the North-East of Italy, bordering with Austria and Slovenia, and it extends from the Adriatic Sea to the Alps. It has around 1,200,000 residents that live in 215 municipalities. The population density is quite low, around 151 inhabitants per square kilometer. The regional health system counts 13 hospitals, grouped in three health districts. Three hospitals, located in the biggest cities of the region (Trieste, Udine and Pordenone) have highly specialized departments and a high case mix complexity, while the others are smaller, with few departments. Every hospital has an emergency department. Moreover, there are six EDs located in structures called “First Intervention Points” (“Punti di primo intervento”, PPI, in Italian) which do not envisage the hospitalization of the patients. A more detailed description of the regional environment and emergency medical service organization is reported in the work of De Ros and colleagues [12]. In FVG the first cases of COVID19 were reported in march 2020 but the first wave was from October 2020 to May 2021. Considering the period 2020–2021 the prevalence rate of positive cases was 1,037 × 100,000, in the middle of the Italian region’s distribution [13]. During the COVID19 pandemic, since October 2020 for all the year 2021, three out of the six first intervention points were closed to relocate the healthcare professional to the facilities more stressed by the pandemic.

This study aims to investigate how the COVID-19 pandemic affected the rate and distribution of non-urgent ED access in Friuli Venezia Giulia, a geographically diverse Italian region. Its mix of urban, rural, mountainous, and coastal areas—combined with notable social and cultural diversity—makes Friuli Venezia Giulia particularly suited for this analysis. We selected variables such as altitude, driving time to EDs, and closure of first intervention points based on prior evidence of their association with healthcare access [14–16], and to assess whether changes in service supply influenced demand during a period of systemic stress.

## Materials and methods

We conducted a retrospective ecological observational study at the municipal level, analyzing admission rates for non-urgent ED visits in Friuli Venezia Giulia during the pre-COVID (2019) and COVID (2021) periods. Non-urgent utilization rates were calculated using the number of ED visits and the resident population as of January 1st, 2019 and 2021, respectively. We calculated unadjusted rates x 1,000 inhabitants.

The primary outcome was the percentage change in non-urgent ED access rates between the two periods, while predictor variables included driving time to the nearest ED, municipal altitude, closure of local first intervention points (used as a binary variable), and the 2019 admission rate.

Patient-level data were extracted from the regional epidemiological data warehouse. We identified patients assigned a “white code” triage classification, which denotes non-urgent status in the Italian healthcare system [17], occurred in the years 2019 and 2021. Each patient’s municipality of residence at the time of admission was used to calculate aggregated rates of non-urgent ED access per municipality.

Driving time to the nearest ED was calculated using the ISTAT (Italian National Institute of Statistics) distance matrix, which reports travel times between municipal centroids [18]. Altitude, expressed in meters above sea level and also provided by ISTAT, was included as a proxy for both accessibility and the degree of rurality.

To assess the impact of first intervention point closures, we classified municipalities with at least 10 residents who accessed one of the three closed facilities in 2019 using a binary variable.

Geographical maps were used to visualize non-urgent ED access rates and their percentage changes between 2019 and 2021. We applied t-tests to assess differences in means and F-tests for variances between the two periods. Following confirmation of a normal distribution for non-urgent ED-utilization rate changes (supplement 1), we performed correlation analyses and t-tests, then estimated a multivariate linear regression model based on the following equation:

$$\begin{gathered}\Delta nuUR = {\beta _0} + {\beta _1}DT + {\beta _2}AL \hfill \\\,\,\, + {\beta _3}CL + {\beta _4}UR19 + \varepsilon \hfill \\ \end{gathered} $$

Where:

- ΔUR = Percentage change in non-urgent ED utilization rate from 2019 to 2021 for the j-th municipality.
- DT = Driving time (in minutes) from the i-th municipality to the nearest ED.
- AL = Altitude (meters above sea level) of the j-th municipality.
- CL = Binary variable indicating closure of a local ED (1 if affected, 0 otherwise).
- UR19 = Non-urgent ED utilization rate per 1,000 inhabitants in 2019 for the i-th municipality.

## Results

**Fig. 1:**
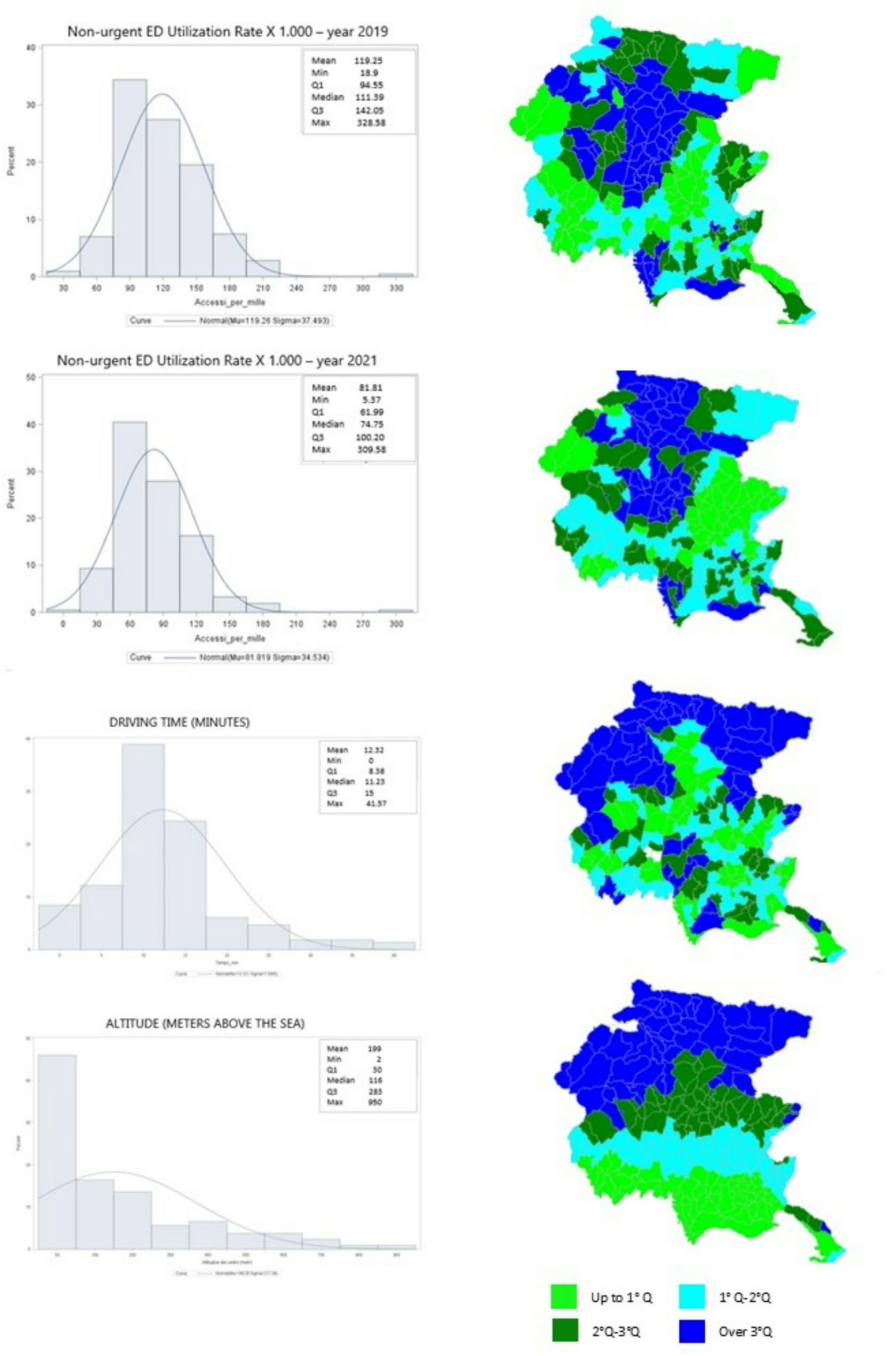
Distribution of: non-urgent ED utilization rate x 1.000 years 2019–2021; driving time (minutes), altitude (meters above the sea) at municipality level

All 215 municipalities of Friuli Venezia Giulia were included in the analysis. Figure 1; Table 1 show the distribution of non-urgent ED utilization rates per 1,000 inhabitants in 2019 and 2021, driving times to the nearest ED, and municipal altitude. The mean non-urgent ED utilization rates were 119 in 2019 and 81 in 2021. This reduction was statistically significant (t-test: 10.78; p-value < 0.0001). However, the difference in variance at the municipal level was not statistically significant (F-test: 1.18; p-value = 0.228).

**Table 1:** Descriptive analysis - Variability and central tendency indexes

Variable	Mean	Std. Dev.	Min	Lower Quartile	Median	Upper Quartile	Max
Non-urgent ED utilization rate 2019	119	37.5	18	94	111	142	328
Non-urgent ED utilization rate 2021	81	34.5	5	61	74	100	309
UR Variation (%)	-31	18.3	-75	-41	-32	-27	49
Driving time (minutes)	12	7.5	0	8	11	14	41
Altitude (meters)	199	217	2	30	116	283	950

Figure 1 illustrates that areas with non-urgent ED utilization rates above the 75th percentile were similar in both years, mainly located in the mountainous and hilly regions in the north and west of the region.

On average, non-urgent ED utilization rates decreased by 31% across municipalities in 2021 compared to 2019. In 75% of municipalities, the reduction was at least 27%.

**Fig. 2:**
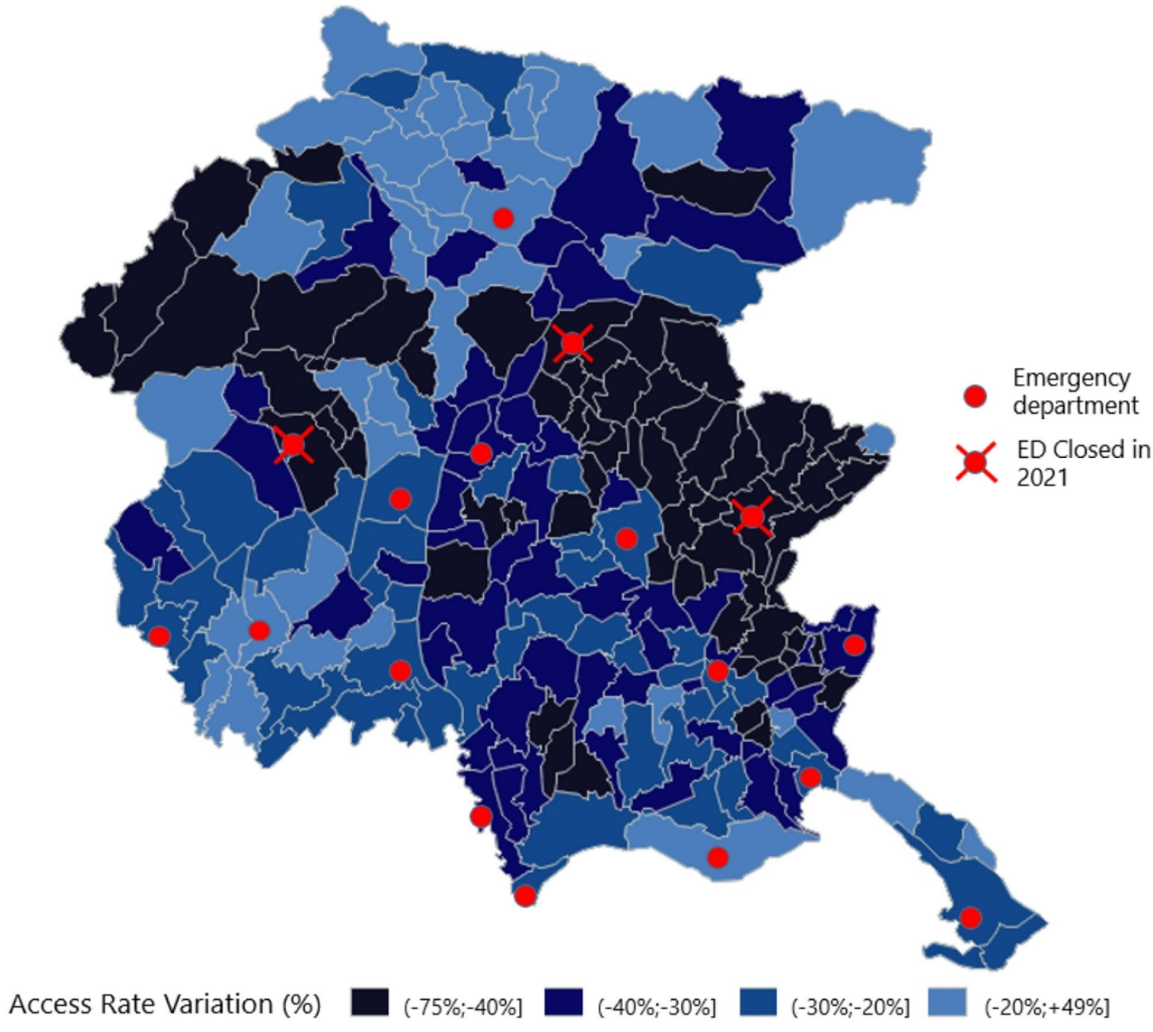
Reduction of non-urgent access rate at municipality level 2021 vs. 2019 and locations of EDs

**Fig. 3:**
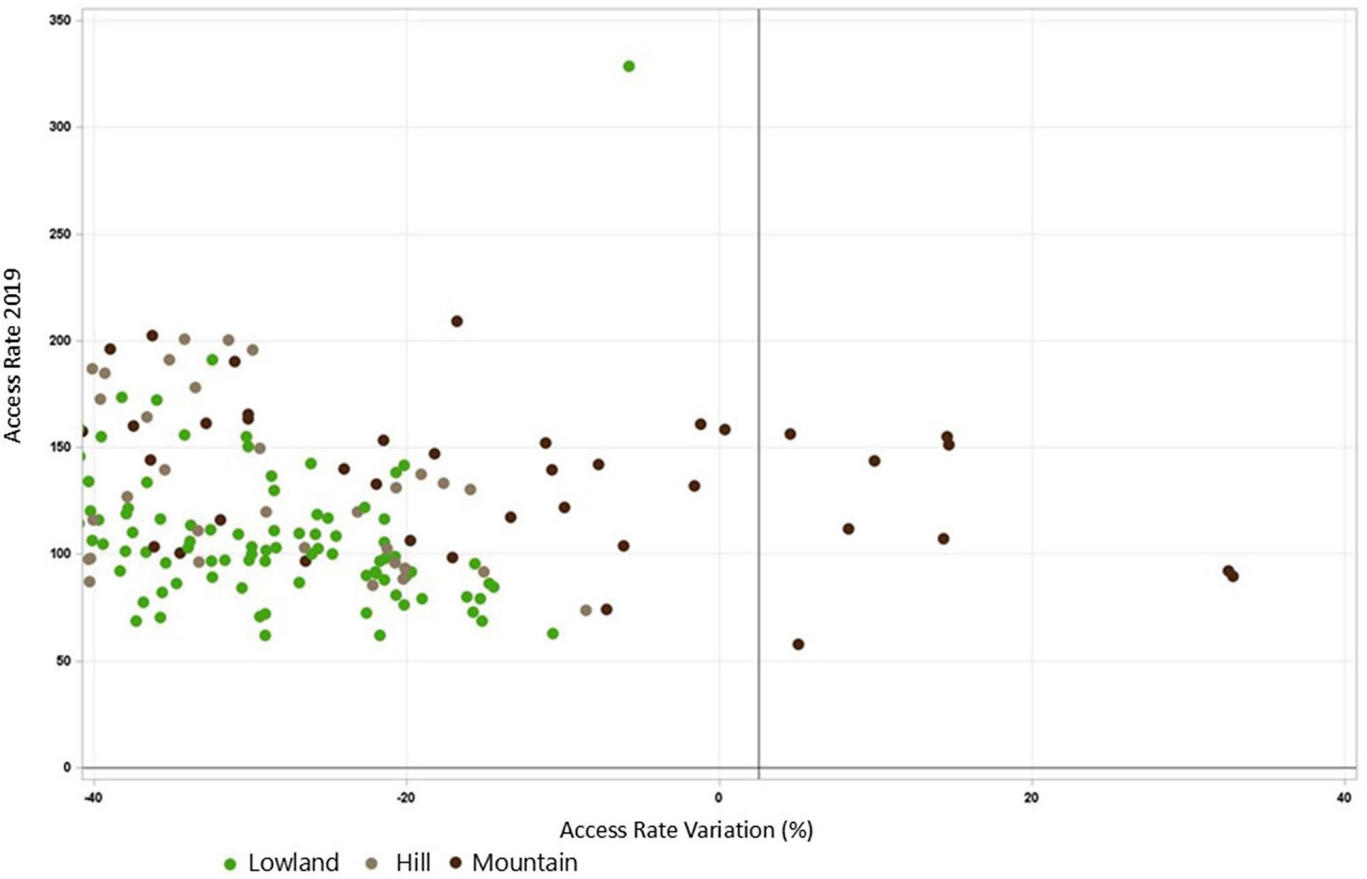
Scatter plot admission rate 2019 vs. admission rate variation per altitude zone (lowland, hill, mountain)

The average driving time to the nearest ED was approximately 12 min, with 75% of municipalities able to reach an ED or first intervention point in under 14 min. Figure 2 provides a regional map of the percentage change in utilization rates (2021 vs. 2019) along with the locations of all 17 EDs, highlighting the three facilities that were closed in 2021. The map reveals that the greatest reductions occurred in lowland and hilly areas near the closed EDs, while mountainous regions experienced relatively lower reductions (Fig. 3).

Correlation analysis showed no significant association between driving time and the percentage change in non-urgent ED access. However, altitude was positively and significantly correlated with the change in access rates (correlation coefficient = 0.25, p-value = 0.001). A t-test comparing municipalities affected by ED closures with those unaffected showed a significantly larger reduction in access rates in the former group (mean reduction of 44% vs. 26%; t-test: 6.93; p-value < 0.0001).

The distribution of municipal-level percentage reductions in access rates was normally distributed (Kolmogorov-Smirnov test = 0.085, p-value < 0.01). Table 2 presents the multivariate linear regression model estimates. All predictor variables were statistically significant at a level of 5%, only driving time was borderline with a p-value of 0.053. The model intercept indicates an estimated average reduction of 37.8%. Specific findings include:

**Table 2:** Linear regression coefficients

Variable	Coef.	Std. Error	Wald Test	*P*-Value
Intercept	-37.8	5.22	52.43	< 0.0001
Driving time (minutes)	-0.53	0.27	3.74	0.053
Altitude (meters)	0.06	0.012	29.64	< 0.0001
ED closure (yes)	-19.69	2.24	76.72	< 0.0001
Non-urgent ED UR 2019	-0.07	0.031	6.20	0.012

Adjusted for the interaction between driving time and altitude (Beta: -0.01; p-value = 0.05)

- An additional 19.69% reduction in municipalities affected by ED closures;
- A further 0.53% reduction for each additional minute of driving time to the nearest ED;
- A 0.06% increase in access for each additional meter of altitude;
- A 0.07% reduction for every additional 1,000 non-urgent visits recorded in 2019.

## Discussion

In 2021, during the second wave of the COVID-19 pandemic, non-urgent ED access in Friuli Venezia Giulia decreased significantly, suggesting a reduction in potentially inappropriate ED utilization. The pandemic served as a natural experiment to investigate some potential determinants of healthcare demand, particularly ED usage.

The overall reduction in ED access between 2019 and 2021 was statistically significant, although no significant change in inter-municipality variance was observed.

Our multivariate analysis identified several key determinants influencing the reduction in non-urgent ED visits during the pandemic. These included:

- Supply-side factors (driving time to EDs, closure of first intervention points);
- Demand-side proxies (baseline non-urgent access rates in 2019);
- Contextual variables (altitude as a proxy for rurality and accessibility).

The data suggest that service supply plays a significant role in shaping non-urgent ED use. Residents of municipalities located further from an ED experienced greater reductions in access rates. The average decrease in non-urgent (white code) ED access across the region was 31%, with reductions of at least 27% in three-quarters of municipalities. A broader analysis in Northern Italy, covering over 15 million ED visits between 2019 and 2023, similarly reported a 41% decrease in white code admissions during the pandemic [19]. In Spain, non-urgent ED use dropped from 20% in March 2019 to just 0.9% in March 2020, even after adjusting for age, sex, and triage scale [20].

Our regression model estimated that the closure of first intervention points contributed to an additional 19.7% reduction in non-urgent ED visits. Importantly, residents in affected municipalities did not appear to compensate by seeking care at nearby EDs. Given the compact geography of Friuli Venezia Giulia, where travel across the entire region requires less than two hours and alternate EDs were within 20 min’ reach, this suggests behavioral shifts rather than access limitations.

Previous studies found similar dynamics in other care domains. Vainieri et al. [21] reported reduced unwarranted variation in elective surgeries in Tuscany during the pandemic, while Bonetti and Melani [22] observed no such reduction in the Province of Bolzano, a mountainous region adjacent to Friuli Venezia Giulia. Our findings are aligned: municipalities with historically higher rates of non-urgent ED use experienced greater reductions, but inter-municipal variation remained largely unchanged.

Interestingly, our analysis distinguished between the effects of driving time and altitude. Even when accounting for travel time, municipalities at higher altitudes exhibited persistently high access rates in both 2019 and 2021. Two possible explanations emerge: (1) reduced access to general practitioners during the pandemic, and (2) a cultural predisposition among mountain residents to prefer hospital-based care.

A limitation of our study is the lack of post-COVID data (2023) on non-urgent ED access, due to changes in triage codification in 2022. Future research should examine overall ED access (both appropriate and inappropriate) to assess long-term variability, especially in relation to the 2023 reopening of the first intervention points closed during the pandemic.

A second limitation of this study lies in the lack of diagnostic-level analysis for individual emergency department visits. While our approach focused on aggregate admission rates and geographic or supply-related determinants, it did not account for the specific clinical reasons behind each non-urgent access. Incorporating diagnostic data could allow for a more granular understanding of behavioral determinants, helping to distinguish between structural factors and case-mix variations. For example, identifying whether reductions affected primarily chronic conditions among the elderly or pediatric low-acuity cases could reveal different patterns of healthcare-seeking behavior and potentially inform targeted interventions.

In conclusion, our study highlights the potential to reduce non-urgent ED utilization through service design. However, this may prove more challenging in mountainous areas, where enhanced primary care accessibility is likely necessary to offset the perceived benefits of hospital-based services.

## Supplementary Information

Below is the link to the electronic supplementary material.

Supplementary Material 1

## Data Availability

Data were obtained from the regional epidemiological repository in aggregated form at the municipal level.
