# Supplementary material for: Effect of COVID-19 and other determinants on the reduction of non-urgent emergency department access in North-East Italy: does supply affect demand?
Source: Res Health Serv Reg. 2025 Sep 1;4:13. doi: 10.1007/s43999-025-00073-1 (PMC12401819; doi:10.1007/s43999-025-00073-1)

**Supplementary materials**

Supplement 1: Scatter plot and correlation coefficients Admission rate Variation vs Altitude and Driving Time. Boxplot Admission rate Variation vs Emergency Departments closure


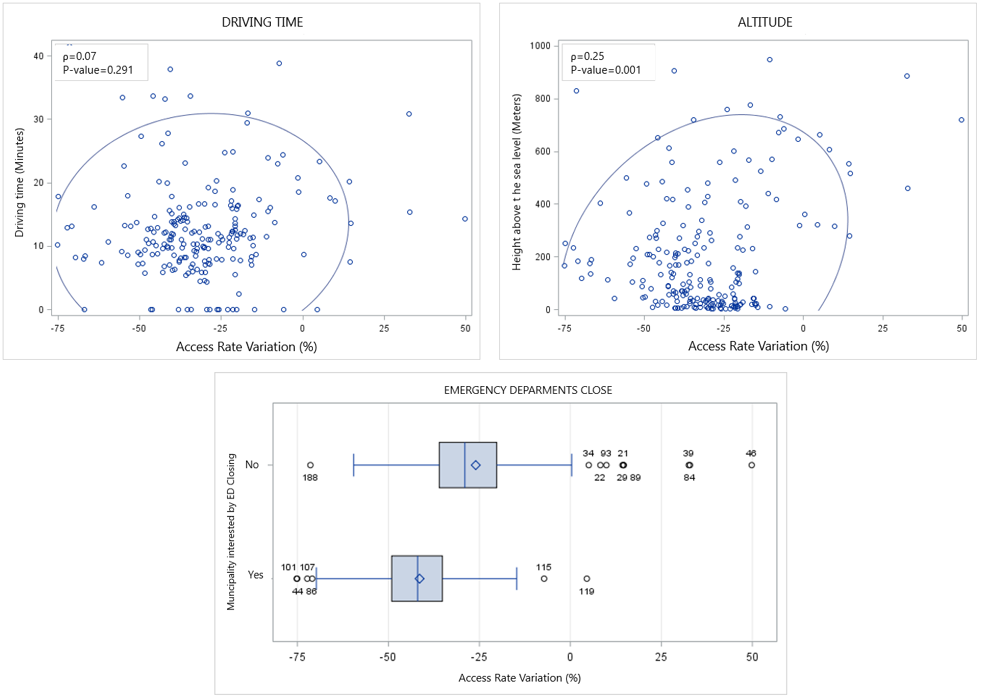


Supplement 2: Scatter plot Admission rate 2019 vs Admission Rate variation per ED closure.


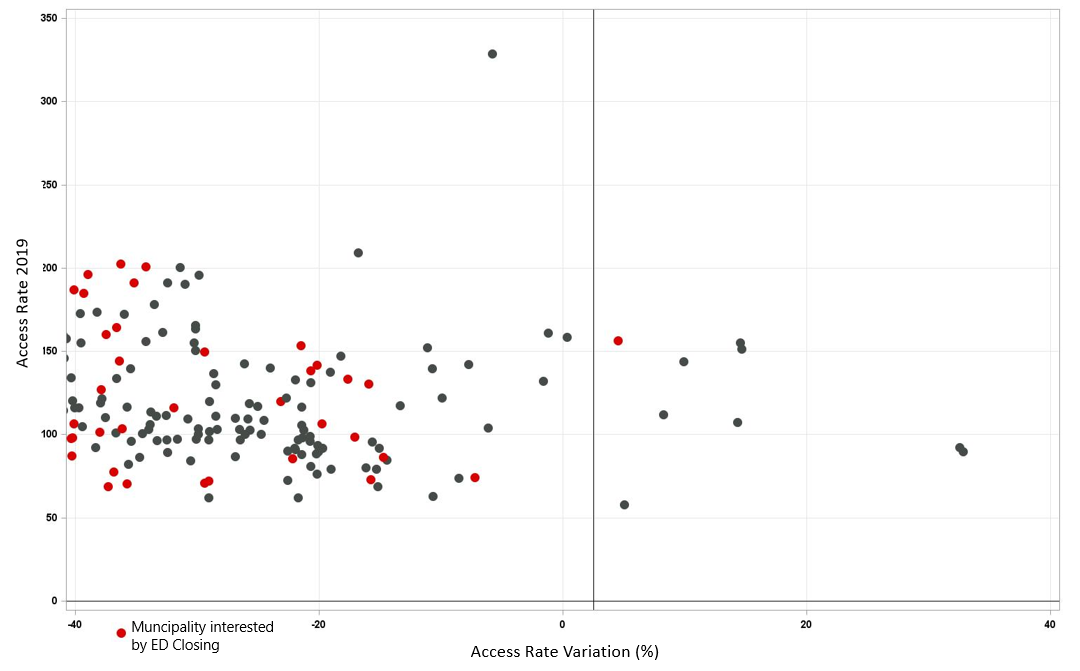

Supplement: Supplementary file 1 — Supplementary Material 1 [file 43999_2025_73_MOESM1_ESM.docx]
